# Application of Human Factors in the Development Process of Immersive Visual Technologies: Challenges and Future Improvements

**DOI:** 10.3389/fpsyg.2021.634352

**Published:** 2021-03-01

**Authors:** Mina Saghafian, Taufik Akbar Sitompul, Karin Laumann, Kristina Sundnes, Rikard Lindell

**Affiliations:** ^1^Department of Psychology, Faculty of Social and Educational Sciences, Norwegian University of Science and Technology, Trondheim, Norway; ^2^School of Innovation, Design and Engineering, Mälardalen University, Västerås, Sweden; ^3^CrossControl AB, Västerås, Sweden

**Keywords:** human factors, augmented reality, mixed reality, virtual reality, training, organizational strategy

## Abstract

This study investigates how Human Factors (HF) is applied when designing and developing Immersive Visual Technologies (IVT), including Augmented Reality, Mixed Reality, and Virtual Reality. We interviewed fourteen people working at different organizations, that develop IVT applications in the Nordic region. We used thematic analysis to derive themes from the interviews. The results showed an insufficient knowledge and application of HF in IVT development, due to the lack of awareness of both scope and significance of HF, resource allocation strategy, market inertia, stakeholder's involvement, standardization of HF application and IVT uses, and technology maturity. This situation could be improved by allocating experts, adjusting organizational strategy to balance resource allocation, training developers and user organizations to raise awareness and to encourage co-creative design and knowledge sharing, create a sense of ownership amongst stakeholders, and ensure the usefulness of the technology to the user's work.

## Introduction

The International Organization for Standardization (ISO) (ISO/IEC, 2018) defines Human Factors (HF) as “scientific discipline concerned with the understanding of interactions among human and other elements of a system, and the profession that applies theory, principles, data and methods to design in order to optimize human well-being and overall system performance.” ISO (ISO/IEC, 2018) also defines “system” as a “combination of interacting elements organized to achieve one or more stated purposes.” This definition in ISO is elaborated on to state that a system is either a “product or as the services it provides” and that system includes “the associated equipment, facilities, material, computer programs, firmware, technical documentation, services and personnel required for operations and support to the degree necessary for self-sufficient use in its intended environment.” From the HF or ergonomics perspective, a system can be more thoroughly defined as Wilson (2014, p. 6):

“A set of inter-related or coupled activities or entities(hardware, software, buildings, spaces, communities and people), with a joint purpose, links between the entities […], and which changes and modifies its state and the interactions within it given circumstances and events, and which is conceptualized as existing within a boundaries.”

Applying HF in early stages of software and technology development has been found to improve the efficiency and productivity of using technology, and consequently organizational performance (Capretz, 2014; Calp and Akcayol, 2015). “Whether a software development project is successful or not depends entirely on the human factor” (Yanyan and Renzuo, 2008, p. 1302). HF has a realistic and profound significance to improve the quality and reliability of software (Yanyan and Renzuo, 2008). HF also has the potential to prevent poor design, as a poor design could have disastrous outcomes if end users are not able to comprehend the tasks they are expected to perform or if the technology is too complex for end users (Sætren et al., 2016). However, the extent and quality of HF application in software design and development did not receive enough attention in early years because research findings have not always been grounded in understanding the nature of design and development and the complexities thereof (Stolterman, 2008).

Traditionally, there has been more focus on the technical aspect than the human aspect of design, but this trend is changing (Sætren et al., 2016). With the rapid development of the technology in the past 30 years, more attention has been paid to curricula and protocols of HF and the importance of users in interacting with computer systems (Ogunyemi and Lamas, 2014). Many tools developed based on research have been used in designing interactive interfaces between humans and technology (Stolterman, 2008) but further improvements can be made if the research becomes even more grounded in better understanding of the design and development practices to support the developers and users (Stolterman, 2008). This is possible through understanding how practice is actually done and how HF is being applied.

This study investigated to what extent HF is being considered and applied during design and development process of Immersive Visual Technologies (IVTs) and what are the reasons behind the sufficient or insufficient HF application. IVTs considered in this research included Augmented Reality (AR), Mixed Reality (MR), and Virtual Reality (VR). Here, we focused on the ISO definition as it is more applicable to a broad range of IVT applications and use cases. The definition adopted reflects on the multidisciplinary nature of HF as it encompasses various fields and focuses on users. It is also the definition that is readily accessible to practitioners who use ISO. HF ensures that when designing tasks, jobs, products, environments, and systems, factors such as physical, cognitive, sociotechnical, organizational, environmental factors are taken into consideration. Three main questions were posed in this paper:

1. What is the status quo of HF in the IVT software development?
2. What are the challenges facing developers in applying sufficient HF?
3. How can HF application for IVT be improved?

This paper aims to build up on the current body of literature on HF and contribute to it by incorporating IVT development process, which is rapidly evolving and expanding technologies as opposed to more traditional technologies. There is a wide range of IVT applications and industries that started to adopt IVT solutions, both in safety-critical and non-safety-critical domains. We interviewed fourteen people from organizations that develop IVT applications in the Nordic region, but they represented a diverse sample of organizations and backgrounds regarding IVT development. We chose the Nordic region as the region has a considerable IVT market (Bezegová et al., 2017) and a tradition of participatory design (Sundblad, 2011). Therefore, HF may already be considered to certain extent in this region.

## Theoretical Background

In the following section we present an overview of the important terms and definitions and the relevant literature about the factors that have been found to influence HF consideration in design and development processes.

### HF Application in the System Design and Development Process

HF application encompasses user analysis (identification of all potential users and their characteristics) (Wickens et al., 2004), task analysis (specification of physical and cognitive actions and the interactions with technology) (Kirwan and Ainsworth, 1992), function analysis (identification of transformations needed by the system to help users with their tasks), environmental analysis (identification of context in of system and user performance) as well as user preference and requirements analysis. It also includes organizational design, which determines the training needs (specification of what, where and whom should receive training) (Salas et al., 2006), as well as equipment redesign and procedural adjustments in the workplace to facilitate user performance.

Usability testing can be done to evaluate user performance and interaction with the system regarding the physical and cognitive abilities and limitations of the users (Wickens et al., 2004). Usability can be evaluated through several criteria, such as the system must be easily learnable, efficient, easy to use and to remember. It must lower the error rate and error recovery must be smooth. The system must be pleasant to use and leave a good user experience (Nielsen, 1993; Brinck et al., 2002; Wickens et al., 2004). It must worth the effort to use (Holzinger et al., 2011). In sum, HF application expands to acceptability evaluation, safety evaluation, and human-technology interaction evaluation (Wickens et al., 2004) such that the system helps users perform their task and achieve their goals.

### Human-Computer Interaction and User-Centered Design

Human-Computer Interaction (HCI) refers to designing to enhance fit between user, computer/technology, and services to improve performance and quality, with respect to the context of use. The term interactive system, as implied in HCI, is defined as the “combination of hardware and/or software and/or services and/or people that users interact with in order to achieve specific goals” (ISO/IEC, 2018). This implies careful consideration of how users interact with technology, physically, cognitively and affectively (Karray et al., 2008) in order to have a user-centered system.

Users-centered or human-centered design “aims to make interactive systems more usable by focusing on the use of the system; applying human factors, ergonomics and usability knowledge and techniques” (ISO/IEC, 2018). This requires ensuring that users are actively participating in the design process and that they receive as much attention as the technical aspects of the system design (Sætren et al., 2016). Therefore, user-centered design is an approach aligned with fulfilling HF requirements in developing new technologies. In fact, the goal of applying HF is to have a user-centered system design (Wickens et al., 2004), which focuses on the software's usability and user experience (Larusdottir et al., 2014), its usefulness and accessibility to the users based on their feedback (Calp and Akcayol, 2015). It emphasizes an interactive way of designing the interface, where needs and expectations of the potential end users and other stakeholders are being met (Pascal et al., 2013).

### HF Considerations in IVT

Designing and developing IVT applications that fulfill users' needs and capabilities are not simple tasks. Although typical design guidelines for desktop-based applications, such as usability heuristics, are still applicable to IVT applications (Murtza et al., 2017), these guidelines are insufficient in terms of informing designers and developers about design choices and their possible trade-offs (Sutcliffe et al., 2019). Here, wrong design choices would not only lead to undesirable performance, but also unhealthy impact on the user. For example, motion sickness is the most commonly reported issue in IVT applications that use head-mounted displays (Aukstakalnis, 2016). Prior research has suggested that motion sickness could occur due to technical issues, such as system's latency (St. Pierre et al., 2015), stereoscopic vision (Keshavarz and Hecht, 2012), limited field of view (Moss and Muth, 2011), and also due to users' characteristics, such as perceptive and cognitive abilities (Stanney et al., 1998). However, it is also important to note that this issue also affects IVT applications differently, for example, users of AR and MR seem to be less susceptible to motion sickness, compared to users of VR (Vovk et al., 2018). All these imply that designers and developers should pay more attention to their users, since IVT applications could influence both their performance and well-being.

### Factors That Influence HF Applications

Despite the vivid importance of HF, it has often been overlooked in the software development, as more emphasis is placed on the technical end of the spectrum of human-technology interactive systems (Capretz, 2014). The limited resources (budget, time, and staff) (Dillon et al., 1993; Rauch and Wilson, 1995; Rosenbaum et al., 2000; Venturi et al., 2006; Bygstad et al., 2008), the lack of end-users involvement (Dillon et al., 1993; Rauch and Wilson, 1995; Rosenbaum et al., 2000; Gulliksen et al., 2004; Bygstad et al., 2008), the organizational culture (Rosenbaum et al., 2000; Iivari, 2006), the complexity of development projects and interorganizational complexity in collaborative projects (Sætren and Laumann, 2014; Milch and Laumann, 2018), as well as the limited knowledge about HF standards and its significance (Sætren et al., 2016) have been mentioned in many studies as the reasons for the suboptimal HF application. In addition, some studies also indicated three more reasons for the suboptimal HF application. Firstly, the homogeneity of end users was found to compromise a culture of questioning about the usability of a new technology implementation (Sætren and Laumann, 2014) and this culture could spread to other stakeholders if they are not exposed to different points of views. Secondly, the late inclusion of HF specialists in the development process could lead to overlooking the evaluation and feedback from HF experts as the prototype may already be produced and there may be limited time, resources and motivations to revise the steps already taken (Rauch and Wilson, 1995). Lastly, the lack of visible impacts of the HF application due to absence of cost-benefit analysis (Rosenbaum et al., 2000; Venturi et al., 2006) and this situation could prevent managements from investing necessary resources for the sufficient HF application. Note that the studies mentioned here are still limited to HF applications in the development of information systems in general, since none of the them specifically investigated HF applications in the development of IVT applications.

## Method

In this section, we present the sample of the participants and how the data were collected and analyzed. We also present an overview of our choices and actions during the data collection and analysis processes.

### Participants

The interviewees were employees in the organizations that develop either AR, MR, or VR applications. The interviewees were mainly working as developers, chief executives, or designers and there was also one research director. The industries that the organizations were targeting varied, such as gaming, education, construction, healthcare, communication, machine building, paper mill, and energy. The organizations provided solutions mainly for training, safety, remote operations, and collaborative work. The organizations worked on the software side of IVT applications and none of them manufactured the required hardware for IVT applications. Most of the organization were start-ups with 4 to 20 employees. Two organizations were established organizations with more than 1,000 employees. Ten organization were based in Finland, two in Norway and two in Sweden.

### Data Collection

A total of fourteen semi-structured interviews were conducted for this study. The semi-structure interview allowed us to get more insight and information from the interviewees' point of views and more flexibility in exploring those views. Interviews were scheduled through emails and carried out from May 2020 to June 2020 by video conferencing calls due to Covid-19 pandemic travel restrictions. Informed consent was obtained prior to interviews. We anonymized and transcribed the interviews verbatim. The interview guide is provided as the Supplementary Material.

### Data Analysis

The interviews were analyzed using thematic analysis, which is “a method for identifying, analyzing and reporting patterns (themes) within data” (Braun and Clarke, 2006, p. 79). It consists of six phases of iterative and repetitive coding and categorizing to derive the themes. The advantages of using this method is its theoretical freedom, its ability to present different perspectives in the data and to provide insight on the main points of the data set (Nowell et al., 2017).

A number of decisions were taken in conducting the analysis. The first one was how the themes will be identified from the interview data. The research question was to a degree guiding the initial coding framework on a broad level, in that we aimed to identifying the context or status quo, the challenges and the improvements that can be made in applying HF. Therefore, at this level, a deductive or top-down approach (Boyatzis, 1998) was taken where coding framework was initially made due to the researcher's interest. However, in order to have a detailed account of all the possible themes and subthemes related to each question, an inductive approach or bottom-up approach (Patton, 1990) was taken to develop themes from the data. Therefore, both approaches were adopted at different stages of analysis.

The second decision was about the level of analysis. In this research, our approach was a progression from semantic level coding to latent coding. “With a semantic approach, the themes are identified within the explicit or surface meanings of the data, and the analyst is not looking for anything beyond what a participant has said or what has been written” (Braun and Clarke, 2006, p. 84). We proceeded to active search for themes and revision of the themes that could help answer the research. For example, what interviewees defined HF to be was initially coded to “knowledge of the term” as part of context/ status quo. In the course of the data analysis, more latent coding was applied which “goes beyond the semantic content of the data, and starts to identify or examine the underlying ideas, assumptions, and conceptualizations and ideologies that are theorized as shaping or informing the semantic content of the data” (Braun and Clarke, 2006, p. 84). For example, parts of the transcript indicated that experienced test users are needed while other parts of transcript text indicated that inexperienced test users are needed. We coded them separately into test user involvement but at later stages it became coded into challenges regarding the identification of the right test user through a systematic method as it reflected on different practices and preferences for selecting test users which latently reflected on different usability criteria of applications and a lack of standard method for user selection.

**Figure 1:**
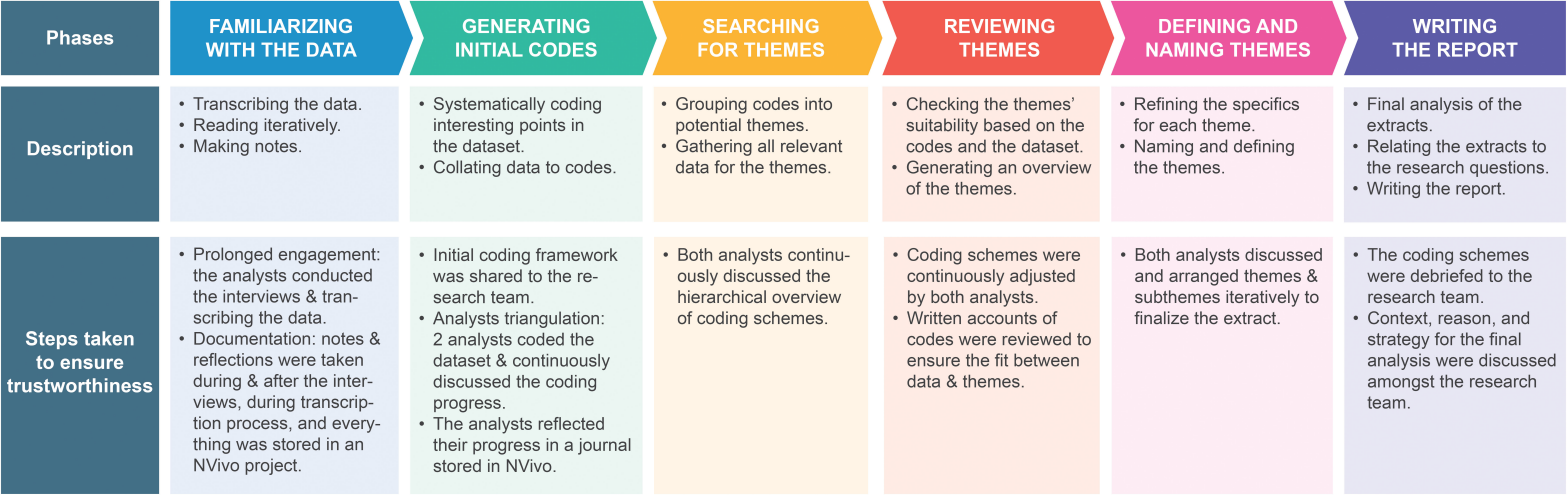
An overview of the six-step analysis and trustworthiness measures per stage.

The third decision was about ensuring trustworthiness, which is equivalent of the validity and reliability criteria in quantitative research (Nowell et al., 2017). Trustworthiness can be ensured through criteria of (1) credibility (the extent to which the researcher has represented the interviewees' view), (2) transferability (being able to generalize the finding by providing the context to the readers so that they can assess to what extent the findings can be applied to their setting), (3) dependability (providing a logical and trackable view of data analysis), and (4) confirmability (showing that the interpretation of the results and the conclusion are data driven) (Nowell et al., 2017). In order to ensure rigor and trustworthiness of the analysis, certain measures were taken at each phase as indicated in the Figure 1 based on the guidelines in Nowell et al. (2017). There were two independent coders that reviewed the codes and discussed them repetitively until consensus was reached. Having different coders and keeping a reflective journal, helped reduce subjectivity caused by researcher's position during the analysis.

Finally, it is worth mentioning that we operated within post-positivistic paradigm. This entails that true reality can only be partly and imperfectly understood (Guba and Lincoln, 1994). We took a constructionist epistemological approach where we seek to understand the sociocultural and sociotechnical context within which experience is produced and reproduced (Braun and Clarke, 2006).

## Results

**Figure 2:**
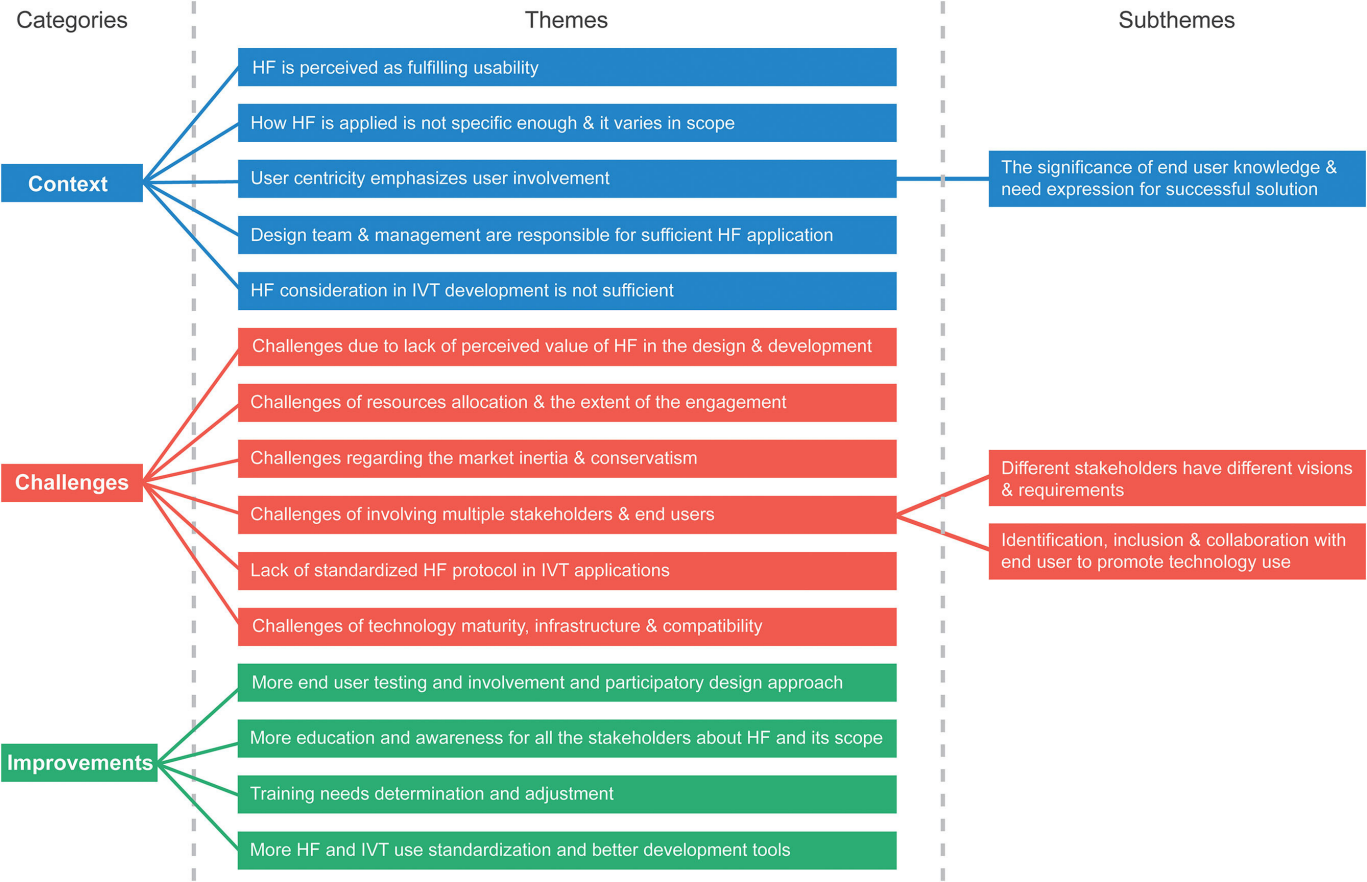
An overview of categories and their respective themes and subthemes.

The aim of this research was to investigate how HF is being applied in IVT software development process. The interviews were analyzed through thematic analysis. We present the results in three categories: (1) the context, that gives information about the status quo of application of HF in IVT development process and its themes, (2) the challenges mentioned explicitly and implicitly in applying enough HF to design and development process, and (3) suggestions for improvements that can enhance HF in IVT development process based on the interview data and its themes. An overview of the themes and subthemes is presented in Figure 2. Some quotes from the interviewees are also provided to support the presented themes and subthemes.

### Context

This category reflects on the current status of knowledge and awareness of HF as a term, its implications and its sufficiency. We first tried to understand whether the interviewees knew about HF. Only five interviewees knew the term HF and what it implied. Majority of the respondents did not know the term itself but could explain how they applied it during development process. If they did not know the term HF, we briefly explained that HF was about considering human abilities and limitations when designing/developing products/services to reduce human error and increase performance and safety. The themes belonging to context are presented next.

#### HF Is Perceived as Fulfilling Usability

The knowledge of the term HF was perceived mainly in terms of (1) developing a system that is simple and intuitive to use, and (2) including users in the design process, addressing the user needs and integrating the best sides of humans and machines to reinforce one another. Knowledge of the term amongst the interviewees ranged from HF being a topic in line with ergonomics and user experience, to an approach that creates a superior unity of human and machine interaction by enhancing the strengths of humans through technology. These were based on the interviewees' own interpretation of the term HF and they were not given our definition of it. Therefore, there was no bias introduced by the interviewers.

#### The Ways HF Is Applied Are Not Specific Enough and It Varies in Scope

Most of the interviewees did not know the term HF, but they were able to explain how they applied what they believed was HF, in the design and development of IVT applications. Some interviewees were in fact talking about why HF was applied rather than how it was applied. For example, they mentioned making a fluid and simple system, ensuring simplicity, making good experience, making a system that allows exploring new concepts and new ergonomic possibilities. One interviewee mentioned that they adopted lean and agile development process to deal with usability needs as they arise. Therefore, certain responses were not specific enough to show how exactly HF is applied in practice. Other interviewees mentioned that they relied on their past experiences and intuition in designing user interfaces, followed by iterative testing or by testing different alternative solutions.

Other responses were more specific, for example, staying close to the end user and the market, involving multiple stakeholders, conducting requirement analysis, system functionality analysis and how to use the system, defining objectives, defining ergonomic needs, defining safety needs, anticipating user needs, incorporating different scenarios, accounting for skills needed to operate the system, and compatibility requirements between the software and the users' platforms. Regarding the methods of collecting data, one interviewee mentioned that they automated HF-related data collection in terms of performance, while others mentioned interviews, workshops, prototype testing and on-site trials. Two interviewees explicitly mentioned having a qualitative and holistic approach of observing and interacting with the users throughout the entire process.

#### User Centricity Emphasizes User Involvement

When it comes to end-user needs, many of the interviewees report having a user-centric approach. One interviewee stated that their goal was to adapt the technology to users' way of working rather than the other way around. This applied to the way end users were already using existing interfaces, for example, swiping the menu options on the displays rather than pressing buttons on the display, and trying to adjust new interfaces to existing habits associated with the older interfaces. Furthermore, market trends, as well as ergonomic and practical requirements seemed to be important factors to consider for building helpful tools for the user.

A central part of the interviews was looking into how end users were involved in the projects in terms of how much influence they had in the development process. Most of the interviewees said they had a user-centric focus in their approaches and that they are in close cooperation with the end users. Whether the customers approached them, or they approached the customers seemed to vary, and for many this could depend on the project. For some it was a co-creative brainstorming session together, while others often started to develop the technology before approaching the customers and without knowing their need and context.

Furthermore, many seemed keen on involving users as soon as possible. End users were involved by giving feedback while developers would evaluate the technical feasibility of the feedback and further give advice to end users on how they can benefit from the technology. One interviewee also mentioned that it was important to build trust with the customers, and that many of the customers wanted to help. Involving the customers would make them feel proud and created a sense of ownership in customers. A subtheme within this theme was about the acknowledgment of the users' knowledge. This subtheme is presented next.

##### The Significance of End User Knowledge and Need Expression for Successful Solution

This subtheme presents the view of the interviewees about their reliance on knowledge and expression of needs by users. One interviewee highlighted the importance of the competencies of their end users and that they were dependent on end users' knowledge in the development project. This was the case for a project where the developers did not have much competency about the user's task. Others also valued having customers with different levels of knowledge with different backgrounds and expertise who provided different points of views and comments. They mentioned that it is easier to work with customers who have a better understanding of the technology, but there was still a need for educating the end users.

#### Design Team and Management Are Responsible for Sufficient HF Application

When the interviewees were asked about who would be responsible for ensuring HF application, they responded that when it came to user interfaces and user experience, the design team is responsible for applying HF to their design. The concept designer was mentioned specifically. The responsibility for making sure that all the development processes make sense and are explicit would be ideally dedicated to the project manager or product owner. When this role is missing, for example, due to budget limitations, then the Chief Technology Officer was mentioned to be responsible for ensuring HF application during development process. One interviewee commented that the entire team needs to be responsible for HF and everyone should ask themselves whether what they are doing would benefit end users and if it made sense. None of the organizations indicated that they had a dedicated HF specialist involved, neither internally nor externally. None of them had a clear division or allocation of responsibility for HF in their organization.

#### HF Consideration in IVT Development Is Not Sufficient

The interviewees were asked if they believed the HF application during development process was enough or not and why. Those who believed it was sufficient indicated that considering budget limit, time pressure and high workload for the project in general, the extent to which they incorporated HF was enough. Therefore, it was a circumstantial “yes.” One interviewee commented that since end users seemed happy and there were no further requests, then there is no need for more HF in the development process. However, they did not mention to what extent they sought feedback after implementing the software. Others believed that it would suffice to be agile and respond quickly to upcoming requests by end users. Others mentioned that it is good, but it can improve if the processes become more standardized and the steps become more explicit. Most interviewees believed that there was not enough consideration of HF in the development process. This was due to a number of issues that were either mentioned explicitly in response to the interview questions or derived from the interview with regard to challenges of applying sufficient HF. The themes that resulted from analysis are presented next.

### Challenges of IVT Development Process That Can Influence Sufficient HF Application

The interviews were analyzed for explicit and implicit indications of the challenges that the interviewees face during development process. These challenges could directly or indirectly influence the HF application. These themes reflect on challenges regarding the lack of awareness of HF's importance, resources, market inertia, multiple stakeholders and end users, lack of standardized HF protocol and challenges of technology maturity. These themes are further explained below.

#### Challenges Due to Lack of Perceived Value and Dialogue About HF in Development

The lack of perceived value of HF was reflected through stronger tendency of developers toward the technical side of human-machine interaction system, as indicated in this quote:

Traditional human factors are trying to focus too much on the human aspects and sacrifice everything from the machine part to comply with the comfort and restrictions on needs of the human, and then there is the opposite. Pure engineering discipline where it's all about the efficiency of the machine and then the human operator

It was mentioned that some developers or engineers may tend to avoid HF and rather create an impressive looking system “I think that's very tempting from an engineer point of view to try to escape some of these steps and say we just make it look fancy.” However, the lack of awareness is also a problem on the customer side. It was mentioned that some customers are not aware of the importance of HF: “They do not understand. They do not look at it in that way […] It's not an issue we can take up.” Another example is “what we actually have a problem within the industry is that we have to make the user (have) a seamless and a good experience. It's not (the kind of) values that they are used to talk about.”

#### Challenges of Resources Allocation and the Extent of the Engagement in the Project

This is one of the most prominent themes mentioned by the interviewees about insufficient HF application. Start-ups and entrepreneurs struggled financially and lacked funding, manpower and time. One of the startups, that had financial limitation, mentioned that they tried to spend their time exploring new solutions and HF could not be prioritized when they had financial struggles, as indicated in this quote:

Budget needs to be there; stuff needs to be there. I think everybody more or less realizes the problem but it's just the business reality that it cannot be solved. It's in the air, people are thinking about it, but there's no way to address the problem at this point.

The customers' limited budget was another reason and that is also the case for short-term projects. It was mentioned that government did not give incentives for innovative technological solutions that could help with obtaining more resources. Another challenge was convincing the customer regarding the importance of HF and approaching those with decision-making power in the right way to convince them to allocate more resources on HF, as indicated in this quote:

For those who then have the resources, I think it's getting to talk to the decision makers in the right way. I mean that perspective, research and reports who could say that this is a good way to do training, that could be very important for us.

Not having enough resources and time to balance the workload and HF requirements was another reason. Another challenge regarding resources is estimating the project costs in advance for innovative solutions where new protocols and many iterative tests with users may be required.

Engagement in the project, in terms of duration, motivation and participation was also mentioned. Long-term projects benefit from more thoroughness in HF, while short-term projects do not, as shown in the statement that “I would say that the longer the project, the more we think about the human factor.” In short-term projects, there is a stricter limit on budget and time. The support after the completion of the project is usually not included for large-scale changes. That could mean that the after-sales support for short-term projects can become an independent project and a new contract would be needed. It was not clear if this served any financial motivation from the interview data.

Long-term projects entail more ongoing relationship and interaction between stakeholders. They can enhance the sense of accountability, the need for building trust, and therefore the motivation to be more thorough with HF. Furthermore, regarding engagement it was also mentioned that the customers should be willing to be more involved in the co-creation process, show interest in learning about the technology and allocating test users and test facilities on site, where developers can run trial tests. The interviewees indicated that this could help developers understand the users' environment and context better. Facilitating on-site presence and communication with the users ensures that developers do not falsely assume that they have all the necessary information needed to design a system or regarding the end users.

#### Challenges Regarding the Market Inertia and Conservatism

This is about market readiness for innovative and groundbreaking technologies, such as IVT, for different use cases and fields. It was mentioned that one of the challenges for developers was the high level of skepticism amongst both decision makers and potential users of IVT applications in traditional and conservative industries. Some development projects are initiated based on a demand by customers, while others are initiated by developers and need to be marketed to the potential customers. The latter may suffer more from market inertia. Much of the work processes and communication methods are old-fashioned and there is a sense of “why change it if it works?” which makes it harder for developers to justify the added value of their solution. This could lead them into compromising HF expenditures to prove the business value of their solutions with less resource expenditures. It can also mean that collaboration and support from market leaders are more difficult to achieve. Less active participation could compromise effective HF on individual, group and organizational levels. In addition to that, the work and communication methods of the developers and end users may be incompatible. Some industries that could utilize remote collaboration platforms are still flying to different locations for the same result of providing support but refuse to change their ways of work, which reflects the role of organizational factor and market inertia:

They have been working the traditional way of buying flight tickets and go to the site for decades, and for them to be able to access their processes to use remote collaboration, it's a big challenge and may take years to be able to fine tune their processes. Even if financially it makes perfect sense, there's just massive inertia in the market.

#### Challenges of Involving Multiple Stakeholders and End Users

This was a prominent theme where people talked about how different parties viewed and appreciated HF differently. There are two subthemes. Each subtheme is presented and elaborated on in the following section.

##### Different Stakeholders Have Different Visions and Requirements

One of the challenges in development process was determining the project objectives for the different stakeholders. Sometimes the enthusiasm level amongst the stakeholders does not balance well with the practical limitations, leading to disappointments. The managers may have one vision, the Research and Development department may want to test the extreme limits of what technology could do and how it can be harvested in years from now, while the operators want a technology that helps them with their task at hand. Satisfying all the various stakeholders is difficult for developers. Furthermore, customers and end users may not have enough knowledge and understanding of technology and its limitations. Another challenge during the development process is making assumptions that the developer has enough information to do usability testing if they go to the site and see the environment. There is still a need for ensuring sufficient information and understanding is obtained. Furthermore, having multiple suppliers means that the support process for end user could be slower, and the end user would need to find other ways to make sure that the work is ongoing. It is a challenge to incorporate these aspects if sufficient information is not acquired from the real end users.

An interesting response reflected on how visionaries in organizations and developers are seeing a future of full automation that the current operators do not see. This was one reason why they did not give enough value to end user's feedback because the users do not see where the future is going. Developers think that, for example, end users who are currently working as operators, will be empowered to become technicians in the future. However, at the same time they are deemed “less aware” of the very future that awaits them and is decided for them:

We think that an operator or a technician will be needed quite a few years ahead but the how they work will change a lot […] we're trying to empower the operators, so they become super technicians. They can still do their great work.

Another factor mentioned was the bias in judgement and evaluation of usability posed by different stakeholders. Managers and decision-makers may not have enough experience to evaluate the usability on behalf of the end users. Meanwhile, some developers mentioned that they did not think that all users are technically “savvy” enough for their feedback to be considered equally, even within the same organization. Another bias mentioned was that software development tools are developed by engineers and for engineers, hence there is not enough emphasis on the users in tools. It is important to emphasize that some of our interviewees showed that they have a different view of HF than others, as can be seen in the following quote:

Software for drawings tend to be made by engineers for engineers. Of course, this means that lots of measuring tools. It's even hurtful to see, as an engineer myself, how little functionality they actually are asking for. They just want their functionality. And they want everything else to be going away. So, they want a very minimalistic user interface. We actually have one of our sayings that the best user interface is no user interface.

Therefore, we want to emphasize that not all engineers and developers have a biased view. Finally, the development of IVT applications was mentioned to be prominently male driven and therefore usability may be compromised for female users with regard to certain features.

##### Identification, Inclusion and Collaboration With End User to Promote Technology Use

Another challenge was defining the “right” end user to be involved in the development process. The interviewees mentioned both challenges of having inexperienced users who may not be familiar with technology and therefore may not give constructive feedback, as well as challenges of having too experienced users whose comments are no longer “pure” feedback. Therefore, it seems that different applications and use cases require different experience levels of users, as seen in the following quote:

I think the challenge always is to find new users, because if the user becomes too familiar with the concept or the feature, then it might not be the good user to test out. So, trying to find like a first-time user, it's a big challenge.

Another relevant quote is:

people are not using extended reality devices currently so much, so we cannot get the real feedback. We can get first feedback from the user, but they are not used to be in the Extended Reality, like gamers who are all the time in the Extended Reality. They can give us the best feedback.

It was mentioned that a wrong user would be the decision makers that choose a solution or device that has low usability but is expensive. In-house testing was considered problematic since real users were not involved as reflected in the following quote:

You are supposed to be wearing this (Head Mounted Display) for several hours at a stretch. This one should be super comfortable and it's not. It has a worse balance than its predecessor. So, it's actually heavy which makes it fall over […] and then it becomes even more uncomfortable. These are the given mistakes. These are things that happen because they only tried this one with managers wearing them, who do not really have the experience. I have some of my people work with it for ten minutes and they say, okay, this one is not as good as I expected it to be. It is a shame because it also costs billions of dollars for the computer hardware.

Another challenge was linked to the volition of use by end users. If they are being watched by supervisors during consulting, they may give false impressions of being happy and content with the solution, in effort to avoid being seen as resistant to change. However, if they struggle with technology use or if they do not perceive it as useful, they may stop using it when they are not being watched by their supervisors.

On-site involvement of end users in the design process can be constrained by safety and accessibility challenges to the user environment. Not every developer is active within safety-critical industries, but for some of the developers, accessing the work site is a challenge as they may need to wait a long time to arrange for visits due to safety concerns, for example, their mere presence and interaction with the end users on site could pose a safety challenge. In addition to that, the safety requirements and challenges for different IVT hardware, such as AR and VR headset are different depending on which environment the user is acting in. The developers need to access and understand the user's context to see how the software is used, transported, stored and what are the safety criteria required on-site to lower the error rate.

#### Lack of Standardized HF Protocol in IVT Applications

Suboptimal standardization of HF requirements and procedures in a wide range of IVT applications, industries and use cases was another challenge to HF application, as mentioned by the interviewees. Some developers are from the gaming industry and some have been involved in more conservative or safety critical industries, where the demands of the product could widely differ as shown in this statement: “I think a lot of things are still in quite intuitive level and they are not too much formal definitions or documented procedures that you follow the steps and life will be beautiful.”

#### Challenges of Technology Maturity, Infrastructure and Compatibility

The responses showed that there is a lack of compatibility between end users' technology platforms and infrastructure and what the developers design. This could compromise user experience. End users may still be using two-dimensional platforms, while the product is designed for three-dimensional platforms. The lack of compatibility poses restriction for design and development, as shown in the following quote:

We are working with industry, which means that a lot of devices, can be very old. The system still has to run on whatever device happens to be found in the pocket of the end user. So, I think that is the biggest thing that creates problems.

In addition, the usual problems of system malfunction or connectivity problem are also present. At the same time, hardware and software evolve rapidly and developers need to continuously update themselves to keep up with this pace. Another problem is that despite the rapid advancement of IVT technology, it is not yet mature enough to be applied widely to its full potential. Furthermore, technical uncertainties and dysfunctional design codes can delay the process, leading to the need for further optimizations in the system.

### Suggested Improvements to Enhance HF in IVT Development Process

This theme reflects on the developers' ideas about how HF application in IVT development process can be enhanced. Three subthemes are identified based on the interviewees' comments. These subthemes are presented next.

#### More End User Testing and Participatory Design Approach

It was suggested that the process of involving end users should be improved. A more diverse range of stakeholders should be involved in development process. The test users should be cross representative of all potential stakeholders to provide a wider range of feedback. This would enhance the understanding of usability for real users, see the following quote:

Currently mostly software users, coders and gamer, like me (are involved in testing) and I think it is a negative side that we do not have so much human testing. We do not know more about the real thing in user computer interface that how people should use the Extended Reality.

Testing should become more incremental during the development process and more interest and initiative from the user organization must be obtained. It was also suggested that the focus must shift from technology to practical advantages for the user, such as usability and learning outcome. The user organizations should be willing to spend more time and dedicate a test site to developers during the project, thus more testing can be done in the users' environment, as shown in the statements that “I think the usual problem is that for example the customer, could be involved more in these applications but they are usually very tight on time” and “having a workspace where we could do the prototyping, that would be good (for testing).” They suggested that more time and resources should be allocated to HF and more specialists should be involved. Furthermore, better communication process and tools are also needed to improve end users' involvement. This can be through remote or digital collaboration platforms.

#### More Education and Awareness for all the Stakeholders About HF and Its Scope

The responses from the interviewees showed that in order to improve HF consideration, there is a need for increased knowledge and awareness of what the term means and how to apply HF in development of IVT, as shown in this statement: “it would be very good if it would be a bit more explicit, a bit clearer how things work and what is done and what is not done.” The interviewees suggested having more resources and more documentation on improving usability for IVTs, as well as educating the developers and customers more on success stories of how larger organizations have benefited from applying HF. The development process would also benefit from staying in a more balanced position in the human-machine interaction spectrum, as shown in the following quote:

It is mentally very hard, and intuitively very hard to be objective in these designs, and actually try to design a system that we cannot give importance to the human. It is very unnatural for us as humans to think that way unless we are for some part brute engineer who thinks about efficiency of the machine. I think to try to stay in the middle and objectively try to focus on how I can reach the system goal in the best possible way, that is a baseline golden kind of thing what we try to follow, and everything derives from that.

More education is needed for both developers and customers, about the importance but also about the breadth of the scope of HF. This means that stakeholders should consider how the technology would fit the broader sociotechnical system and how different stakeholders will be affected by it. More communication is needed to spread awareness and to help both the market and the end users to be more open toward innovative solutions.

#### Training Needs Determination and Adjustment

There should be more emphasis on identifying training needs and outcomes using technology, as shown in this statement:

You also must keep in mind who are the target groups, what is the learning target, what should be the learning or training outcome and then you talk to technologists. We often talk about technology. We should talk a lot more about learning outcome, learning targets and choosing the right method to reach those targets. And in some perspectives the VR is very good, and in other perspectives the VR is not good at all.

The way in which the training is implemented, however, seems to vary a lot among the companies. Different ways of offering training were mentioned by the different interviewees, including web-based e-learning packages with virtual training, in-built training as the basic part of the application, game-based learning platform, YouTube-video tutorials, demo sessions, and five interviewees also mentioned about visiting the customers and either spend the whole day or some hours to show them how to use and how to set up the application. Meanwhile, for some interviewees the training was not deemed necessary. This variation in training methods could be explained by what was the solution that was offered and how complicated it was. The companies also vary in their opinions on how sufficient they think the training is. One interviewee stated that the goal was to empower the user enough so that training was not necessary. Despite the differing view on training that was presented, most interviewees shared the view that there is a need to learn and adapt to the new technology for the years to come, for instance, some have experienced that there was a low tolerance for technical issues and difficulties among the users. They believed that user organizations should be more responsible to determine training needs for their users and should offer it themselves. They also believed that end users should be more trained on how to use IVT and be more up to date with the technology.

#### More HF and IVT Use Standardization and Better Development Tools

This theme reflects on the need of more standardization of how to apply HF and how to promote it amongst developers. More knowledge sharing is needed, as shown in the following statement:

I actually have no idea how other AR studios function, or what their process is. It could be quite nice to maybe have AR production process discussion with our colleagues and see how they do things and what they found to work best. I do not know who should do it. I think everyone should do it.

They also suggested having HF specialists involved in the process, or a dedicated person that can focus only on HF. It was also suggested that there is a need for standardization of the technology hardware and software itself and having a standard way of using, thus end users could learn easily. Having less controllers and more intuitive way of using IVT was mentioned, as indicated in this statement: “we are hoping that virtual reality will either evolve to be without controllers, or there will be some standardization.” In addition to better and more standardized processes, the development tools should be improved as well. One interviewee mentioned that the development tools should also become more standardized and should be further refined. Better manuals need to be developed as well for both developers and potential end users.

## Discussion and Future Implications

The aim of this paper was to understand how HF is applied in IVT development process. The context represented the Nordic region and the interviewees represented a diverse group of developer organizations of different sizes and market niches. IVT is a fast-evolving technology with varied use cases and potentials in different industries. Three research questions were investigated through conducting interviews, to understand the context of HF consideration in the development process of IVT, the challenges and the improvements that could be made.

The results of the thematic analysis of the interviews revealed that the majority of the interviewees across the fourteen organizations were not familiar with the term HF, its constituent elements, and its scope. This could be because the term HF is defined differently based on the discipline and educational background (Capretz, 2014), and it can be rather abstract and quite broad in scope (Laumann et al., 2018). Furthermore, this knowledge may not be accessible to everyone outside academia or certain industries (Shorrock and Williams, 2016). HF encompasses many disciplines, many steps and categories of analyses that are not always distinct from one another. Furthermore, the term involves individual level, group level, organizational level and sociotechnical level factors (Laumann et al., 2018). However, most of the current HF literature are at the individual level (Laumann et al., 2018). If most developers are not aware of HF, they cannot apply it. However, when the interviewees explained how they applied HF, we could see that some of them had a better understanding of the scope of the HF than others. Therefore, although it is difficult to expect that a developer applies HF at every level, it can be expected that they are aware of the significance and breadth of HF. Nevertheless, regarding how they applied HF in their work, they mentioned about adopting an iterative process, involving users and carrying out usability testing. One interviewee mentioned that they adopted agile development approach to deal with usability needs. However, Larusdottir et al. (2014) found that an agile approach was not always as responsive to users' needs as assumed and it was not always aligned with user-centered design. The current state of HF is marked by the lack of sufficient knowledge and awareness of the term and its application. Furthermore, not every interviewee had a clear understanding of the standards that should be followed regarding HF.

ISO offers standard processes, but it could be that not every practitioner has access to ISO or that they are not required to abide by it. It can be that due to the vast application and requirement for customization, the standards are not as detailed and customized. It can also be that some developers have gained their expertise through experience rather than through education, or those working in smaller organizations, may not know about all the standards. In the future research, we should ask about the extent of awareness and adherence to the ISO recommendations.

We compared the definitions and perceptions of the interviewees with the definitions provided by ISO. This comparison showed that the definitions and the application of HF from the perspective of the interviewees was perceived to be about enhancing the interaction between human and the elements of the system to improve the overall system performance. This was consistent with the first part of the ISO definition of the term HF. However, not everyone was aware of the term, and therefore, also not aware of the tasks of an HF professional, as indicated in the second part of the ISO definition. Furthermore, the perception of the term “system” was different among interviewees. Some interviewees referred to system as the technological product or the service that the human-technology interaction could offer, while others named more elements of the system, such as “multiple stakeholders” that are involved. The term system in ISO is defined as a “combination of interacting elements organized to achieve one or more stated purposes” (ISO/IEC, 2018) and other definitions mentioned in section Introduction, provided a more holistic approach to the term system, compared to the ISO definition. Therefore, we believe that it is not surprising that the perceptions of the term system are varied amongst interviewees. There is also a difference between the HF expert's view of the term system compared to some of the designers and developers. The focus and the scope are narrower for developers and designers, but they are not wrong, nor inconsistent with the ISO definition of the word system. One of the improvements could therefore be to clarify the term “system” and what it entails for different stakeholders involved.

In the field of HF, the term system is perceived to extend to the sociotechnical context and is seen as a more holistic term. However, as a system is composed of its components that have a relation to each other and interact, a software can be regarded as a system as well. Therefore, the difference in the disciplines, and knowledge and awareness of the other disciplines and their paradigms, plays a role in how “system” is defined and what this word brings to the mind of a HF expert vs. a software developer. This was also reflected in the interviews where the word system was more often used to refer to the technology itself or the user-technology interaction, rather than the broader scope. This highlighted difference is also another reason why HF is not fully appreciated for its breadth in practice.

The interviewees' implications about perceiving the term usability were quite thorough. ISO defined usability to be “extent to which a system, product or service can be used by specified users to achieve specified goals with effectiveness, efficiency and satisfaction in a specified context of use” (ISO/IEC, 2018). In general, we believe that the interviews mentioned many criteria that covered this definition, such as simplicity, learnability, intuitiveness, ergonomic design, availability amongst others in their interviews, showing that the term is more familiar than the term HF. Regarding the user or human-centered design, ISO defined the term to be an “approach to system design and development that aims to make interactive systems more usable by focusing on the use of the system; applying human factors, ergonomics and usability knowledge and techniques.” The application of HF is implied but despite the fact that most interviewees mentioned to be user-centric, few of them knew the term HF, which has the goal of creating user centered systems. The interviews also showed that they applied user-centered design in different ways and the challenge of selecting the right user was also mentioned. The definition of user centered design is more thorough but perhaps too general. This implies that in practice, there will be more deviations and customizations than what ISO suggests. This is also because the challenges of design and development, per project, are different. Therefore, it is not surprising to see differences between ISO and interpretations or practices by the software developers. It again reflects back on the importance of converging research and practice (Stolterman, 2008), and to see how and why guidelines and practices could differ.

Furthermore, as HF needs to be further clarified, IVT taxonomy, application and use cases need to be clarified, standardized and more explicit. There is a plethora of use cases across industries currently for IVT. This poses a serious challenge for introducing one unique ISO guideline for all the stakeholders in this field. However, creating a knowledge bank and promoting cross-communication can promote knowledge sharing and accessibility. This can become a challenge in today's competitive market and patent-driven technology development. However, industries can start by clarifying what is the usability criteria for different industrial use cases. Concepts such as realism, immersion and their trade-off (Jerald, 2015) need to be more researched and standardized.

In this section, we present further suggestions, in addition to what was suggested by the interviewees, on what could be done to improve HF consideration and application in IVT development process.

### HF Should Be Allocated to Experts at Each Level of HF

HF responsibility at each level should be allocated to experts in that particular level. For example, designers and developers deal mostly with individual level, such as user need analysis, task analysis and function analysis (Laumann et al., 2018). At this level, they can benefit from expert opinions on these matters. In this case, it will be the real end users. If an HF specialist would be recruited in the project at this stage, he or she would have to know about physiology, cognition and individual limitations of the user. At the next level, group and organizational levels, other experts need to be involved. Decision makers, such as managers and organizational scientists or HF experts with organizational design knowledge, can be included to re-design work tasks, work environment, scheduling design, balance resources, facilitate communication, training and user participation (Vicente, 2003; Wickens et al., 2004). This consideration is important because “a lack of consideration for organizational culture and standard practice is another problem of transferring HCI values, and practices to [software engineering] processes” (Ogunyemi and Lamas, 2014, p. 4). This would require adjustment in organizational strategies on both developer and customer sides (Rauch and Wilson, 1995; Venturi et al., 2006). Wilson (2014) explained that some organizations appoint their HF experts to one level, such as design, development, safety, training for example, while other organizations may appoint the HF experts to all the levels. However, if there are not enough HF experts in large organizations, they may either become marginalized or they may blend in to perspective of that level at the cost of losing the more holistic perspective (Wilson, 2014). Therefore, we believe that for each HF level, people who have expertise in addressing issues and necessities on that particular level should be involved. They also need to cross-communicate, thus educating one another across levels in order to cover the entire scope of HF in sociotechnical system (Venturi et al., 2006). We would like to add that HF experts have a broad base of knowledge that should not be limited to only one specific level. They have the ability to see the links and apply the knowledge across different levels and this expertise should be made use of. The literature suggested involving a HF specialist (Sætren et al., 2016). The problem is that if the term HF is unclear, the expectation of what a HF specialist must do is also unclear. The supposed areas of expertise for an HF specialist is too wide. It includes physiology, cognition, software development, organizational management and sociology. There should be more knowledge about what could be expected from HF specialists and how they can be supported by other experts in organizations.

Another factor to consider is that recruiting an HF specialist should not stop developers' need for more education about HF. The need to learn about HF and applying it in design, should become a standard procedure, not an add-on feature for developers. In addition, hiring an HF specialist will not automatically address the issue if the given role is more of a consulting role, where the HF specialist does not have significant influence in the development process (Iivari, 2006) and the decision making. We believe that a dedicated multidisciplinary team, with expertise related to each level working with HF experts, and a dedicated project manager could be a practical approach to improve HF application.

### Organizational Strategy Can Balance Resource Allocation

Most interviewees indicated HF was not applied enough due to limited resources. One of the interviewees who gave this reason also mentioned how they continuously explored new opportunities. This requires time which is also a form of resource. Both start-ups and established organizations mentioned the lack of resources to result in the lack of HF. If every organization struggles with allocating resources to HF, the problem may lie with prioritization or balancing the resources, rather than the lack of it. Therefore, the organizational strategy in both developer and user organizations need to be adjusted to prioritize HF. However, not every organization took a passive approach. There were two organizations that stated how they stayed very close to the end user and focused on bringing value to the end user from the start of the design and development process. Therefore, we believe that adjusting organizational strategy and communicating that HF is an inherent and essential part of development, not only internally but also externally to the customers, will encourage resource allocation to HF (Rauch and Wilson, 1995; Venturi et al., 2006).

The management will also need to take lead in balancing the resource allocation, negotiating with the customers about the cost vs. benefits of HF and showing the success stories of how it can promote long-term use and benefit. One of the interviewees mentioned that everyone knows HF should be emphasized more, but nobody wants to talk about it, which is aligned with the findings in Rauch and Wilson (1995), Bygstad et al. (2008). Managers can bring this topic into organizational discussions and encourage this approach by investing in it.

### Further Education and Training for Both Developers and User Organizations and Co-creative Designing

Some interviewees blamed the lack of knowledge of customers for compromised HF. However, they did not mention to what extent they actually included HF clauses in their contract negotiations. If the customer is well-informed about the importance of HF, they would not knowingly neglect it if they were aware of financial payback in the long term. Nevertheless, the different customers in different industries must also educate themselves. They must try to understand both potentials and limitations of the technology if they want to empower their employees through human-machine interaction solutions. Furthermore, they must not assume that a developer or an engineer can understand every aspect of the end user's task and context. They should actively promote interactive and co-creative innovation and dedicate a multidisciplinary team to encourage early-stage communication and information exchange. This leads to cross-education. The shared knowledge will further facilitate future technology implementations.

The clarification of usability criteria can also help with selection of the right users to be involved in the development process. Once that is known, the developer and user organizations can create dedicated multidisciplinary groups where the right end users are identified and their needs are prioritized, and then communicated to the project team. This can help aligning the visions of different stakeholders which was one of the challenges of developers. The bias in judgments and design can be amended by diversity and end-user centricity as opposed to customer- centricity. The inclusion of the right end user and the support of the project team will create a sense of ownership and accountability for the co-creation process (Boivie et al., 2003). This can enhance volition of use and brings long-term benefits for the IVT solution in the user organization.

### Reflections on the Potential Training Structure and Scope to Enhance HF

We propose that training should become more standardized for both user organizations and developer organizations. It should perhaps begin by showing the value of the HF consideration by displaying how the industries, that have applied HF, have benefited from doing so. It can show success stories and failure stories to capture attention in both target audiences. Training in HF is usually associated with preparing the end users mentally and physically for different job situations (Wickens et al., 2004). The end users training has often been discussed, but we would like to add the importance of training that targets attitude about giving honest feedback, meaning that they should learn to give salient feedback and do not hold back due to conformity. It also aligns with promoting a questioning culture amongst end users where a healthy level of resistance, and being critical, can enhance safety through enhancing HF (Sætren and Laumann, 2014).

The developer training should bring awareness about the psychological principles that influence both end users and developers and how to account for these principles. Developers are prone to the same psychological and cognitive propensities as anyone else and these cause a bias in judgement in design. They should be trained to be aware of the occurrence of biases, spot and act against dysfunctional patterns that could arise due to these propensities, such as confirmation bias and sunk-cost fallacy (Vicente, 2003). Confirmation bias is about seeking information that proves our point of view and dismissing what opposes our point of view (Wason, 1959; Nickerson, 1998). Sunk-cost fallacy is about continuing to invest in a lost cause, in hope of recuperating it (Arkes and Blumer, 1985). When, a minimum viable product is developed and time and budget has been invested already, non-confirmatory feedback from HF specialist and/or end users may not be received well. It may introduce new and non-confirmatory information that may be unwelcome. Prevention can be done through training on spotting and reducing biases (Nickerson, 1998), which could be considered as part of the organizational training. Furthermore, conducting more empirical research and actively look for information that is disconfirming can help overcome biases (Stacy and Macmillan, 1995).

It was mentioned in the literature that the software issues were often not physical factors, but they were psychological factors (Vicente, 2003). It can be because quantitative data on physical side of usability and design are prioritized over the psychological side of usability and design. More training on different methods of data collection, both quantitative and qualitative and more openness to different methods should be provided to developers. It can enhance developers' appreciation of not only the hard factors of design but also the soft factors (Wickens et al., 2004), such as the adjustment of work schedule and duration of using an IVT software with regard to human limitations, or the design of the work environment to mention a few (Vicente, 2003). This requires a training on adopting system thinking where both humanistic and mechanistic views are considered to understand the complexity of a system. This is a shift in paradigm from mechanistic or reductionist view into a more human-technology approach that is more characteristic of our modern world complex systems (Wickens et al., 2004; Oliveira et al., 2020).

At the organizational level, training should bring awareness to how resources are allocated and what that implies in the day-to-day prioritization of tasks under time limit. This expands to awareness of the incentive structure. Managers should be trained to be more mindful of their indirect communication through resource allocation. They should be trained to start a dialogue about HF-related topics that may not be given their duly place in both intra and inter-organizational dialogues. They should be trained on conducting HF cost-benefit analyses at the onset of new technology implementations. This will enhance organizational performance in the long run.

A shift in paradigm is needed. Most safety critical-industries, where HF was researched in, had traditionally adopted a mechanistic view, where focus is primarily on hardware and software, but have shifted toward a more human-technology approach and it must happen before it is too late (Vicente, 2003). We argue that this shift should expand to all other industries involving an interaction between human and technology.

## Limitation and Future Work

As mentioned in section Participants, the interviewees came from the organizations that worked on the software-side of IVT applications. With respect to hardware, most interviewees were limited to what they or their clients already had in their possession, what fitted their existing platforms, what they were familiar with and/or what fits within the client's budget (see sections Challenges of Resources Allocation and the Extent of the Engagement in the Project and Challenges of Technology Maturity, Infrastructure, and Compatibility) This resulted in less discussion of the hardware side of IVT applications in the interviews, even though what the software could do is highly dependent on the chosen hardware. Selecting the intended hardware based on HF considerations is also an equally important consideration, since hardware can also have direct implications on users' performance and experience when using IVT applications (Aukstakalnis, 2016). It is known that there is a correlation between technology specifications and users' sense of presence, although not all technology specifications had equal effects on users' sense of presence (Bowman and McMahan, 2007; Cummings and Bailenson, 2016). When the budget for IVT development is limited, it is advised to put more resources on hardware that offers stereoscopic vision, wider field of view and better tracking, since these specifications have higher influence on users' sense presence (Cummings and Bailenson, 2016). Nevertheless, it would also be interesting to conduct a similar study with more emphasis on the hardware side of IVT applications.

## Conclusion

The aim of this paper was to understand how HF is being applied in IVT development process across diverse industries. Three main questions were asked for this purpose: what is the status quo of HF in IVT software development? What are the challenges facing developers in applying sufficient HF? And how can HF application for IVT be improved? The answers to these questions helped to understand that HF application in IVT process is not well-understood by developers in its full scope, but it was partly practiced through usability testing and early end-user involvement in iterative design process. However, it is not applied enough. The challenges regarding the suboptimal HF application expanded from lack of awareness and knowledge of HF, to resource allocation, market inertia and multiple stakeholders that were involved in the process and their conflicting visions, biases, needs and selecting the right users to be involved in the process. Lack of standardization of HF protocol and IVT use, as well as the challenges regarding technology maturity and compatibility were also mentioned. Improvements suggested are as follow: (1) HF should be allocated to expert at each level of HF, (2) organizational strategy can balance resource allocation, and (3) further education and training for both developers and user organizations and co-creative designing. This will enhance knowledge sharing regarding usability criteria, helping to identify the “right” users to be included at each level of HF, aligning the visions of different stakeholders, overcoming bias through diversity of stakeholders and specificity of participants and expert at each level of HF and its stages to ensure a user centricity.

## Data Availability Statement

The anonymized raw data supporting the conclusions of this article will be made available by the authors, without undue reservation.

## Ethics Statement

The studies involving human participants were reviewed and approved by Norwegian Centre for Research Data. The patients/participants provided their written informed consent to participate in this study.

## Author Contributions

MS and TAS were responsible for planning and designing the study, reviewing literature, and collecting the data. MS and KS were responsible for analyzing the data. MS and TAS were responsible for writing the manuscript. KL was responsible for provision of funding and planning the study. KL and RL were responsible for providing feedback and editing the manuscript. All the authors approved the submitted version.

## Conflict of Interest

TAS was employed by company CrossControl AB. The remaining authors declare that the research was conducted in the absence of any commercial or financial relationships that could be construed as a potential conflict of interest.
