# Supplementary material for: Application of Human Factors in the Development Process of Immersive Visual Technologies: Challenges and Future Improvements
Source: Front Psychol. 2021 Mar 1;12:634352. doi: 10.3389/fpsyg.2021.634352 (PMC7957067; doi:10.3389/fpsyg.2021.634352)
Supplement: Supplementary file 1 [file Data_Sheet_1.docx]

Supplementary Material – The Interview Guide for the Semi-structured Interviews

1. BACKGROUND INFORMATION

(Can you introduce yourself and confirm that you agree to the content of informed consent form)

1. Could you shortly describe the organization you work at?
2. What is your current position?
3. What is your educational background (shortly)?
4. How long have you been working at the current organization?
5. When was the first time you professionally developed an AR/VR application?
6. WORK AND WORKPLACE
7. If it can be disclosed, can you describe your typical workday?
   - What are your main responsibilities?
8. What is your current position? How is the social environment at your workplace?
9. Experience with new technological developments at your company
10. Can you tell me about how you design/develop new technologies at your company?
    - Who makes the decision to design/develop new technologies?
    - Where do the ideas for the design/development come from?
    - What is the process for designing/developing new technologies like?
    - How often do you design/develop new technologies?
11. AR/VR for Industry
12. What industries or projects do you mainly develop AR/VR solutions for?
    - What do you think about it?
    - How do you think this new technology would change how your target end users work?
    - In what way do you think the use of AR/VR would make their work safer and more efficient?
13. HUMan factors
14. Have you heard about human factors? Are you familiar with it? Could you describe it?
15. Do you think you are responsible for including human factors in the development process? If not, who is responsible for it?
    - How are the end users involved in the development process?
    - What human factors do you consider when developing VR/AR solutions?
    - How do you do the human factor analysis?
    - Do you think there is enough consideration for human factors in the development of product? Why/why not?
    - What do you think should be done to improve that?
16. In your opinion, what kind of negative effect that AR/VR applications could cause to end users?
    - Did you receive complaints about motion sickness and nausea?
17. COMMUNICATION AND FEEDBACK
18. How do you communicate with clients before you start to develop a new product?
    - How do you spread information about new technological developments in the industry?
    - How do you usually communication to your current and potential clients about new developments?
    - What is the collaboration like usually?
    - How do you communicate with clients during the negotiation phase about the features and the pricing of the product?
19. How do you communicate with clients during the development phase?
20. How do communicate with and support the clients after delivering the product?
    - Do you provide any training for them?
    - How is the training set up?
    - Is the training enough?
    - What are the most common questions and problems that you receive?
    - How did you resolve the problems?
21. How do you think the development and use process can be improved in the future?
